# Cultural Familiarity and Individual Musical Taste Differently Affect Social Bonding when Moving to Music

**DOI:** 10.1038/s41598-020-66529-1

**Published:** 2020-06-22

**Authors:** Jan Stupacher, Maria A. G. Witek, Jonna K. Vuoskoski, Peter Vuust

**Affiliations:** 1 Center for Music in the Brain, Department of Clinical Medicine, Aarhus University & The Royal Academy of Music Aarhus/Aalborg, Aarhus, Denmark; 2 Institute of Psychology, University of Graz, Graz, Austria; 3 Department of Music, University of Birmingham, Birmingham, United Kingdom; 4 RITMO Centre for Interdisciplinary Studies in Rhythm, Time and Movement, Department of Musicology & Department of Psychology, University of Oslo, Oslo, Norway

**Keywords:** Human behaviour, Social behaviour, Psychology

## Abstract

Social bonds are essential for our health and well-being. Music provides a unique and implicit context for social bonding by introducing temporal and affective frameworks, which facilitate movement synchronization and increase affiliation. How these frameworks are modulated by cultural familiarity and individual musical preferences remain open questions. In three experiments, we operationalized the affective aspects of social interactions as ratings of interpersonal closeness between two walking stick-figures in a video. These figures represented a virtual self and a virtual other person. The temporal aspects of social interactions were manipulated by movement synchrony: while the virtual self always moved in time with the beat of instrumental music, the virtual other moved either synchronously or asynchronously. When the context-providing music was more enjoyed, social closeness increased strongly with a synchronized virtual other, but only weakly with an asynchronized virtual other. When the music was more familiar, social closeness was higher independent of movement synchrony. We conclude that the social context provided by music can strengthen interpersonal closeness by increasing temporal and affective self-other overlaps. Individual musical preferences might be more relevant for the influence of movement synchrony on social bonding than musical familiarity.

## Introduction

Social bonds have long been associated with enhanced mental and physical health and well-being^1^. How well we connect with another person depends, among others, on our cultural background, individual preferences, and the context of a given situation. Music provides a unique social context by introducing temporal and affective frameworks, which increase behavioural synchrony and emotional harmony. Individuals collectively synchronize their movements with rhythmical features of the music on a temporal scale down to milliseconds. This type of temporal framework provides a shared understanding of a group’s behaviour by increasing the predictability of others’ movements, for example in dance. In addition, by listening to the same music, people share common contextual information and establish joint attention, ultimately building up a collective affective experience driven by cooperation and affiliation. Following Phillips-Silver and Keller^2^, we refer to the synchronization of movements as *temporal* social entrainment and to the sharing of emotional experiences as *affective* social entrainment.

*Temporal* social entrainment is observable in the tendency to synchronize movements and behaviour in everyday interactions such as walking^3,4^, chair rocking^5^, and joke telling^6^. These and other expressions of interpersonal movement synchronization have been shown to promote *affective* social entrainment in form of affiliation, cooperation, and altruistic behaviour^7–11^. The prosocial effects of temporal entrainment seem to be particularly strong when moving together with music^12–15^, suggesting that music adds a powerful social context to interpersonal interactions. Stupacher and colleagues^14^ used a social entrainment video paradigm to investigate how moving together with music or a metronome affects affiliation. They found that with music, but not with a metronome, the likeability of another virtual person was lower when this person was walking out of phase and oneself in phase, as compared to the other way around (other person in phase and oneself out of phase). This interaction suggests that although both music and metronome provide a stable auditory timekeeper, music might add a more meaningful context to the social situation. With more familiar and more enjoyed music, this unique social context might become even more meaningful.

All over the world, music is predominantly performed *in* groups^16,17^ and oftentimes *for* groups to induce bodily movements and to emotionally unite people^16^. In social interactions, music facilitates coordination by increasing the predictability of another person’s behaviour and mood. Accordingly, it has been argued that human musicality might have evolved to facilitate social living^18,19^. In line with this argumentation, various studies demonstrated that the abilities and preferences for social entrainment are learned early in human development^13,20,21^, for a review, see^2^.

By being exposed to specific musical structures in our everyday life, we become musically enculturated and acquire culture-specific musical knowledge from an early age^22^. Whether we recognize a musical rhythm or perceive a clear beat in a rhythm depends, among other things, on the amount of exposure to music with similar rhythms. In-culture biases have for example been shown for the recognition of music^23^ and rhythm perception^24–27^. Thus, what is considered as an adequately tight level of temporal social entrainment with music might depend on individual preferences and the cultural background of a listener, dancer, or musician^28^.

Accordingly, affective aspects of social entrainment might also be influenced by musical taste and enculturation. Musical preferences of mutual friends are more similar than those of randomly paired persons^29^. Additionally, in Afro-Brazilian Congado – a ritual with multiple musical ensembles playing at the same time while moving through the town – groups of the same community are more likely to entrain than groups of different communities^30^. Even without active movement or interpersonal interactions, listening to music from a specific culture can increase the implicit preference for facial pictures of people from that culture^31^. The last mentioned studies provide some evidence that musical preferences and enculturation are important factors in social interactions with music. However, detailed information about how an individual’s familiarity with and enjoyment of specific types of music are related to social entrainment is scarce.

## Experimental Overview

**Figure 1:**
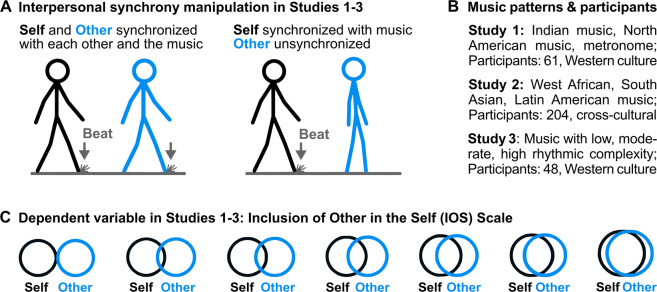
Design of Studies 1–3. (**A**) Interpersonal movement synchrony was manipulated by using the social entrainment video paradigm. Participants watched two walking stick figures and imagined that one of the figures represents themselves and the other figure represents an unknown person. Left panel: Example of one frame of a video with synchronized virtual self (black) and virtual other (blue). Both figure’s steps are aligned with the quarter beat of the musical pieces. A stylized dust cloud additionally marked the temporal position of the beat. Right panel: Example of one frame of a video with the virtual self in synchrony with the quarter beat of the music and the virtual other out of synchrony. (**B**) Different musical stimuli used in the three studies and participant samples. (**C**) Adapted Inclusion of Other in the Self scale (IOS^32^) used in all three studies. Participants rated the interpersonal closeness between virtual self and other on a continuous slider presented below the 7 circle combinations.

Figure 1 provides an overview of the design of Studies 1–3, which used a social entrainment video paradigm similar to Stupacher and colleagues^14^. In the videos, two figures were walking side by side. One figure represented a virtual self and the other figure a virtual other person.

The temporal aspect of social interactions was manipulated by movement synchrony: while the virtual self always moved in time with a musical context, the virtual other moved synchronously or asynchronously (Fig. 1A). In Studies 1 and 2, the musical context was provided by instrumental music typical for different topographical regions. In Study 3, we manipulated the rhythmic complexity of the musical context by creating low, moderate, and high levels of syncopation.

The affective aspect of social entrainment was operationalized as ratings of interpersonal closeness between virtual self and other measured by the Inclusion of Other in the Self scale (IOS, Fig. 1C)^32^. In Studies 1 and 2, participants additionally rated the familiarity with and enjoyment of the music. In Study 2, participants also rated the beat clarity of the music.

We hypothesized that the interpersonal closeness to the virtual other would be stronger when moving synchronously compared to moving asynchronously – i.e., a main effect of movement synchrony. Additionally, we expected that the difference of interpersonal closeness to a synchronous compared to asynchronous virtual other would be larger with more familiar and more enjoyed music – i.e., interaction effects. This would suggest that for highly familiar and highly enjoyed music, movement synchrony is more relevant for social bonding than for less familiar and less enjoyed music.

## Results

### Study 1

Sixty-one Western participants rated the interpersonal closeness to and likeability of another virtual person in a social entrainment video paradigm (Fig. 1A). While the virtual self was always walking synchronously with the beat of the musical context, the virtual other was walking either synchronously or asynchronously. The patterns of the musical context were either culturally familiar, culturally unfamiliar, or a metronome.

First, we analysed the whole dataset in two separate ANOVAs, one for *inclusion of other in the self* and one for *likeability of the virtual other* (see supplementary material). Results indicate that interpersonal closeness and likeability ratings were higher with music compared to a metronome. Furthermore, closeness and likeability ratings were higher with familiar compared to unfamiliar music.

For the main analysis, we fitted two separate linear mixed effects models to a dataset only including responses to videos with music. With this dataset, we investigated the effects of *synchrony*, *musical pattern* (familiar vs. unfamiliar), and *enjoyment of the music* on the two dependent variables *inclusion of other in the self* (IOS) and *likeability of the other* (LIKE).

#### Dependent variable IOS

**Figure 2:**
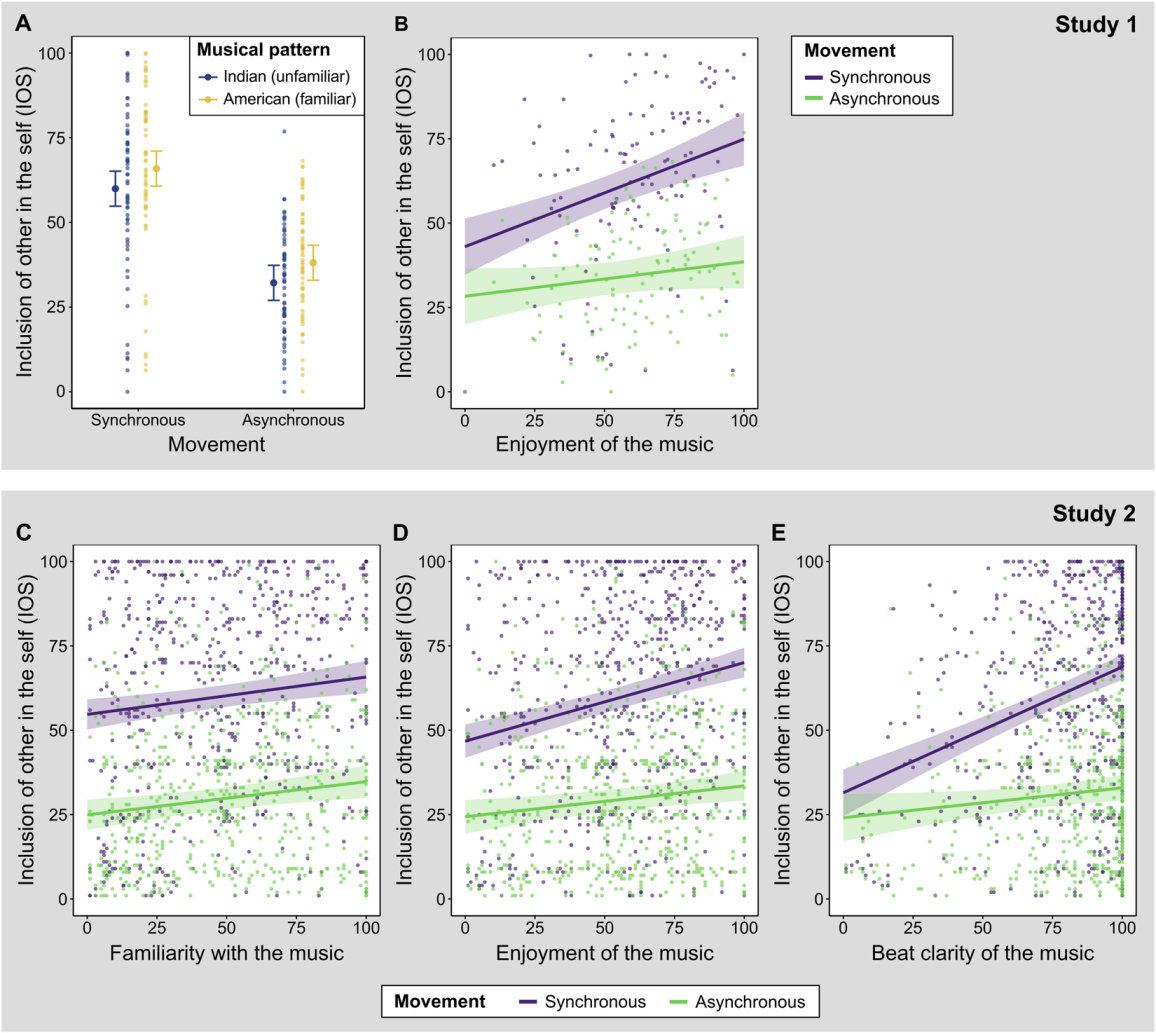
Results of Studies 1 and 2. Data points and model-predicted values of interpersonal closeness, as measured by IOS, in Study 1 (panels **A** and **B**) and Study 2 (panels **C–E**). Error bars / shaded areas represent 95% confidence intervals. Data points and predicted values for each musical pattern of panels **B–E** are depicted in Supplementary Figures S1 and S2.

Detailed model comparisons for the dependent variable *inclusion of other in the self* are listed in the upper panel of Table 1. Including terms for the main effects of *synchrony* and *musical pattern* improved the model fit (Fig. 2A, for pairwise comparisons see Supplementary Table S1). The model fit further improved with a term for the interaction between *synchrony* and *enjoyment of the music*. This interaction indicates that when participants enjoyed the music more, their ratings of interpersonal closeness increased more strongly with a synchronized virtual other compared to an asynchronized virtual other (*t*(179) = 3.11, *p* = 0.007, Supplementary Table S1; Fig. 2B).

**Table 1:** Nested mixed effects models for the two dependent variables *inclusion of other in the self* (IOS) and *likeability of the virtual other* (LIKE) investigating the effects of the independent variables *synchrony*, *musical pattern* (i.e., cultural familiarity), and *enjoyment of the music*. Every model includes the random effect (1 | participant). The Akaike information criterion (AIC), Bayesian information criterion (BIC), marginal *R*^2^ (variance explained by fixed effects only), and conditional *R*^2^ (variance explained by fixed and random effects) are provided. *χ*^2^ and *p* values refer to model comparisons to the previous model (unless stated otherwise) using likelihood ratio tests. Null model: IOS ~ (1 | participant). Best fitting models are marked in bold letters.

Dependent variable: IOS; Independent variables: Synchrony, Musical Pattern, Enjoyment of Music						
Model	AIC	BIC	Marginal *R*^2^	Conditional *R*^2^	Improvement in Model Fit	
					*χ*^2^(1)	*p*
IOS Null Model	2235	2246		0.338		
IOS ~ Synchrony	2059	2073	0.310	0.750	178.52	<0.001
IOS ~ Synchrony + Musical Pattern	2047	2064	0.324	0.767	14.35	<0.001
IOS ~ Synchrony × Musical Pattern	2049	2069	0.323	0.766	0	0.999
IOS ~ Synchrony + Musical Pattern + Enjoyment	2035	2056	0.356	0.770	13.17°	<0.001°
**IOS ~ Synchrony × Enjoyment + Musical Pattern**	**2028**	**2052**	**0.364**	**0.781**	**9.61**	**0.002**
**Dependent variable: LIKE; Independent variables: Synchrony, Musical Pattern, Enjoyment of Music**						
**Model**	**AIC**	**BIC**	**Marginal** ***R***^**2**^	**Conditional** ***R***^**2**^	**Improvement in Model Fit**	
					*χ*^**2**^**(1)**	*p*
LIKE Null Model	2097	2108		0.299		
LIKE ~ Synchrony	2007	2020	0.210	0.577	92.87	<0.001
LIKE ~ Synchrony + Musical Pattern	1977	1994	0.260	0.642	31.66	<0.001
LIKE ~ Synchrony × Musical Pattern	1978	1999	0.260	0.642	0.36	0.551
**LIKE ~ Synchrony + Musical Pattern + Enjoyment**	**1934**	**1955**	**0.398**	**0.655**	**44.38^**	**<0.001^**
LIKE ~ Synchrony × Enjoyment + Musical Pattern	1935	1960	0.399	0.656	1.06	0.304

°As compared to model IOS ~ Synchrony + Musical Pattern.

^As compared to model LIKE ~ Synchrony + Musical Pattern.

#### Dependent variable LIKE

Detailed model comparisons for the dependent variable *likeability of the virtual other* are listed in the lower panel of Table 1. The comparisons showed the best fit when adding main effects of *synchrony*, *musical pattern*, and *enjoyment of music*, but no improvement in fit when adding the interaction term *synchrony* × *enjoyment*. This means that participants rated the *likeability of the virtual other* higher with more enjoyed music, independent of movement synchrony. (*t*(239) = 7.08, *p* < 0.001, Supplementary Table S1).

### Study 2

We conducted a second study with a culturally diverse participant sample (N = 204) consisting of a main study, which was preceded by two pre-studies for musical stimulus selection (see supplementary material). The main study used a social entrainment video paradigm (Fig. 1A), to investigate the effects of musical familiarity and enjoyment on interpersonal closeness with a synchronously or asynchronously walking virtual other person. In an additional exploratory analysis, we tested the effect of beat clarity on movement synchrony. Participants rated the *inclusion of other in the self* (IOS) between virtual self and virtual other walking with instrumental music typical for the broad regions West Africa, South Asia, and Latin America.

We modelled IOS as a function of the following effects in linear mixed effects models: *synchrony*, *musical pattern*, *music rating* (either *familiarity*, *enjoyment*, or perceived *beat clarity*), and the interaction between *synchrony* and *music rating* (Table 2). Model comparisons revealed a main effect of *synchrony* and *musical pattern*. Adding the interaction term *synchrony* × *musical pattern* did not improve the fit (Table 2). For *familiarity with the music*, the best fitting model additionally indicated a main effect of *familiarity*, but no interaction between *familiarity* and *synchrony* (Fig. 2C). This means that interpersonal closeness was generally higher with more familiar music (*t*(1201) = 3.70, *p* = 0.001; see Supplementary Table S2 for pairwise comparisons). For *enjoyment of the music*, the best fitting model indicated an interaction between *enjoyment* and *synchrony*. This means that higher *enjoyment of the music* was associated with stronger increases of interpersonal closeness with a synchronized virtual other compared to an asynchronized virtual other (*t*(1015) = 3.49, *p* = 0.003, Supplementary Table S2; Fig. 2D). For *beat clarity*, the best fitting model indicated an interaction between *beat clarity* and *synchrony*. This means that higher *beat clarity* was associated with stronger increases of interpersonal closeness with a synchronized virtual other compared to an asynchronized virtual other (*t*(1015) = 6.14, *p* < 0.001, Supplementary Table S2; Fig. 2E).

**Table 2:** Nested mixed effects models for the dependent variable *inclusion of other in the self* (IOS) separately investigating the effects of the three independent variables *familiarity with the music*, *enjoyment of the music* and perceived *beat clarity*. Every model includes the random effect (1 | participant). The Akaike information criterion (AIC), Bayesian information criterion (BIC), marginal *R*^2^ (i.e., variance explained by fixed effects only), and conditional *R*^2^ (i.e., variance explained by fixed and random effects) are provided. *χ*^2^ and *p* values refer to model comparisons to the previous model (unless stated otherwise) using likelihood ratio tests. Null model: IOS ~ (1 | participant). Best fitting models are marked in bold letters.

Dependent variable: IOS; Independent variables: Synchrony and Musical Pattern						
Model	AIC	BIC	Marginal *R*^2^	Conditional *R*^2^	Improvement in Model Fit	
					*χ*^2^ (*df*)	*p*
Null Model	11663	11679		0.306		
IOS ~ Synchrony	11096	11116	0.247	0.603	569.56 (1)	<0.001
**IOS ~ Synchrony + Musical Pattern**	**11089**	**11120**	**0.251**	**0.607**	**10.83 (2)**	**0.004**
IOS ~ Synchrony × Musical Pattern	11088	11129	0.252	0.608	4.89 (2)	0.087
**Dependent variable: IOS; Added independent variable: Music Familiarity**						
**Model**	**AIC**	**BIC**	**Marginal** ***R***^**2**^	**Conditional** ***R***^**2**^	**Improvement in Model Fit**	
					***χ***^**2**^ **(*df*)**	***p***
**IOS ~ Synchrony + Musical Pattern + Familiarity**	**11077**	**11113**	**0.261**	**0.607**	**13.68 (1)°**	**<0.001°**
IOS ~ Synchrony × Familiarity + Musical Pattern	11079	11120	0.261	0.607	0.11 (1)	0.745
**Dependent variable: IOS; Added independent variable: Music Enjoyment**						
**Model**	**AIC**	**BIC**	**Marginal** ***R***^**2**^	**Conditional** ***R***^**2**^	**Improvement in Model Fit**	
					***χ***^**2**^ **(*df*)**	***p***
IOS ~ Synchrony + Musical Pattern + Enjoyment	11058	11093	0.273	0.612	33.26 (1)°	<0.001°
**IOS ~ Synchrony × Enjoyment + Musical Pattern**	**11048**	**11088**	**0.276**	**0.617**	**12.13 (1)**	**<0.001**
**Dependent variable: IOS; Added independent variable: Beat Clarity**						
**Model**	**AIC**	**BIC**	**Marginal** ***R***^**2**^	**Conditional** ***R***^**2**^	**Improvement in Model Fit**	
					***χ***^**2**^ **(*df*)**	***p***
IOS ~ Synchrony + Musical Pattern + Beat Clarity	11049	11085	0.281	0.615	41.59 (1)°	<0.001°
**IOS ~ Synchrony × Beat Clarity + Musical Pattern**	**11014**	**11055**	**0.292**	**0.628**	**37.19 (1)**	**<0.001**

°Improvement in model fit as compared to model IOS ~ Synchrony + Musical Pattern.

### Study 3

**Figure 3:**
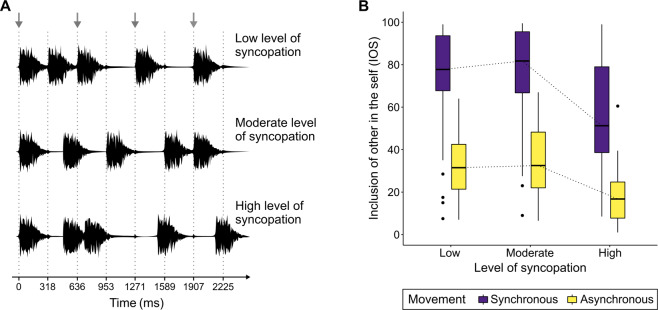
Stimuli and results of Study 3. (**A**) Waveforms of one bar of the three different musical stimuli with low, moderate, and high levels of syncopation. The dotted grey lines represent the eighth-note level at a tempo of 94.4 beats per minute. The grey arrows on top mark the strong metric positions at the quarter-note (beat) level. In the stimulus with low syncopation, four of five piano chord onsets fall on the strong metric positions, compared to two in the moderately syncopated and one in the highly syncopated stimulus. The smaller peaks represent a soft hi-hat sound, which was marking the eighth notes. (**B**) *inclusion of other in the self* ratings for videos with synchronously or asynchronously moving figures accompanied by musical stimuli with three different levels of rhythmic complexity (low, moderate, and high levels of syncopation). Boxes represent the interquartile range (IQR); whiskers represent the lowest values within 1.5 * IQR of the lower quartile, and the highest values within 1.5 * IQR of the upper quartile; dots represent outliers; dotted lines represent the connections between the medians (center line).

We used a social entrainment video paradigm (Fig. 1A) to directly manipulate and investigate the effect of beat clarity on social closeness in synchronous and asynchronous movement interactions. Beat clarity was manipulated by adjusting the level of syncopation of the musical context in the videos (Fig. 3A). The dependent variable was *inclusion of other in the self* (IOS).

In both movement conditions, synchronous and asynchronous, IOS ratings were higher when the *syncopation level* of the context-providing music was low and moderate compared to high (Fig. 3B, Table 3). No significant differences were found between low and moderate *levels of syncopation*. A comparison of the mean IOS ratings of all three *levels of syncopation* revealed a main effect of *synchrony* with higher IOS ratings for synchronous movements (*Z* = 5.88, *p* < 0.001, *r* = 0.60, N = 48).

**Table 3:** Pairwise comparisons (Wilcoxon signed-rank tests) between *inclusion of other in the self* ratings in videos with different *levels of syncopation* (low, moderate, and high), N = 48. The Bonferroni-corrected critical p-value is 0.05/6 = 0.0083.

	Low vs. moderate syncopation			Low vs. high syncopation			Moderate vs. high syncopation		
	*Z*	*p*	r*	*Z*	*p*	r*	*Z*	*p*	r*
Synchronous movement	−1.70	0.089	0.17	5.43	<0.001	0.55	5.77	<0.001	0.59
Asynchronous movement	−0.96	0.340	0.10	5.47	<0.001	0.56	5.51	<0.001	0.56

*Effect size r = Z/sqrt(N×2) with N = 48.

The difference of IOS ratings between synchronous movement and asynchronous movement tended to be larger with moderate *levels of syncopation* compared to high *levels of syncopation* (*Z* = 2.12, *p* = 0.034, *r* = 0.22, N = 48, Bonferroni-corrected critical p-value 0.017). No significant effects were found for the comparison of IOS difference values between low and high *levels of syncopation* (*Z* = 1.68, *p* = 0.094, *r* = 0.17, N = 48) and between low and moderate *levels of syncopation* (*Z* = −0.20, *p* = 0.842, *r* = 0.02, N = 48).

## Discussion

In a series of three experiments, we showed that participants felt closer to a virtual other person when self and other were moving synchronously with music. We additionally investigated how familiarity, enjoyment, and beat clarity of the musical context affect interpersonal closeness. With more familiar music, interpersonal closeness increased. Importantly, this increase was similarly strong for synchronous and asynchronous movements of the virtual other. In contrast, enjoyment and perceived beat clarity of the music were associated with strong increases of interpersonal closeness when moving with a synchronized virtual other, but only weak increases of interpersonal closeness when moving with an asynchronized virtual other. This finding suggests that with less enjoyed and less rhythmically clear music, movement synchrony is less relevant for social bonding than for highly enjoyed and rhythmically clear music.

In line with previous research, we demonstrated that interpersonal synchronization of movements on a millisecond scale increases affiliation^7–11^. The prosocial effects of movement synchronization might stem from an increased self-other overlap, which can be understood as a “phenomenon whereby an observer engages a state similar to that of the target via activation of the observer’s personal representations for experiencing the observed state”^33^. As perception-action links are considered crucial for self-other overlaps, social understanding, and empathy^33–36^, interpersonal synchrony of movements might facilitate the ability to put oneself in another’s shoes – on a simulated motor level and on an affective empathic level^35,37^. Furthermore, in line with Stupacher and colleagues^14^, our findings demonstrate that active movement is not a prerequisite for social evaluations of interpersonal synchronization, suggesting that the perception-action links relevant for social understanding are also utilized when watching or imagining a social situation. How do these evaluations of interpersonal movement interactions change in social contexts with more and less enjoyed and more and less familiar music?

Studies 1 and 2 indicate that when the music was more enjoyed, social closeness increased strongly with a synchronized virtual other, but only weakly with an asynchronized virtual other. This interaction is in line with our hypothesis and demonstrates that preferences for certain types of music are an important factor for social evaluations. Individuals use another person’s musical preferences to infer their personality^38^ and have more similar musical preferences with their friends compared to other persons^29^. With less enjoyed music, movement synchrony between self and other might be less relevant, because the music provides a less meaningful social context. With highly enjoyed music, however, the social context might become more meaningful. In this situation, synchronized movements of another person might not only match one’s temporal but also affective expectations, as the musical preferences between oneself and the other person are interpreted as congruent. In contrast, asynchronized movements of another person might not only reduce temporal social entrainment, but also affective social entrainment because the preferences of oneself and the other person are interpreted as incongruent.

Contrary to enjoyment of music, familiarity with the context-providing music did not interact with movement synchrony, as shown in Studies 1 and 2. This means that different levels of familiarity did not affect the relation of interpersonal closeness with a synchronously compared to asynchronously walking virtual other. However, in Study 2, higher musical familiarity was associated with higher interpersonal closeness in general. The same effect of familiarity was found in Study 1 when looking at a model only including terms for movement synchrony and familiar vs. unfamiliar musical patterns. Importantly, with an added term for enjoyment of the music, the effect of familiarity was nonsignificant. This suggests that in Study 1 enjoyment of the music better explained the ratings of interpersonal closeness than familiarity with the music.

Although enjoyment of the music in a social context might be more important for interpersonal closeness than familiarity with the music, familiarity still plays a central role. Listening to a familiar song with another person provides a basis for shared knowledge. Even four to six year-old children would prefer to befriend another child who knows songs that they are familiar with compared to a child who does not know these songs, indicating sensitivity to shared cultural knowledge^39^. Similarly, in adults, shared knowledge and shared preferences about bands, books, or movies, endorse interactions with another person^40^. The main effect of familiarity on interpersonal closeness might be a result of these prosocial effects of shared knowledge. Independent of interpersonal movement synchrony, the more familiar the music, the more one might appreciate the sharing of one’s knowledge, positively influencing social bonding.

In addition to familiarity and enjoyment, participants of Study 2 rated the beat clarity of the context-providing music. Exploratory analyses revealed that, similar to the effect of enjoyment of music, increased beat clarity was associated with more widely separated ratings of interpersonal closeness with a synchronously compared to asynchronously walking virtual other. When the perceived beat clarity was higher, interpersonal closeness increased strongly with a synchronized virtual other, but only weakly with an asynchronized virtual other.

To validate this effect of beat clarity, Study 3 directly manipulated rhythmic complexity by introducing musical contexts with different levels of syncopation. When compared to high levels of syncopation, low and moderate syncopation levels in both movement synchrony conditions resulted in higher interpersonal closeness. This finding might relate to the inverted U-shaped relationship between syncopation, pleasure, and body movement: rhythms with moderate levels of syncopation are more enjoyed and induce more movement than rhythms with low and high levels of syncopation^41,42^. This inverted U-shape can also explain why differences in interpersonal closeness between synchronous and asynchronous movements were higher for videos with moderate syncopation levels compared to high syncopation levels. Matthews and colleagues^41^ showed that stimuli with moderate syncopation levels are enjoyed most and high syncopation levels least. Assuming that the current auditory stimuli, which were selected from Matthews and colleagues, evoked the same pattern of enjoyment, our finding confirms the *synchrony* × *enjoyment of the music* interactions found in Studies 1 and 2. Comparable to Studies 1 and 2, the current results suggest that higher enjoyment of musical rhythms was associated with more clearly separated ratings of social closeness between synchronous and asynchronous movement interactions. Additionally, the higher differences in social closeness between synchronous and asynchronous movements with moderately compared to highly syncopated rhythms support the *synchrony* × *beat clarity* interaction found in Study 2.

Higher beat clarity enables more accurate temporal predictions of musical events. In social interactions with music, precise temporal predictions sharpen evaluations of temporal social entrainment making it easier to assess whether oneself and others are moving in synchrony with the music or not. Thus, the ability to perceive a clear beat in music is central for assessing temporal social entrainment, which in turn is influencing affective social entrainment. Converging evidence suggests that the predictions of *what* musical event will happen *when* are key aspects for the experience of pleasure and the rewarding effects in music^43–46^, cf.^47^. Similar effects might occur for predictions of other persons’ behaviours.

Our findings of Studies 2 and 3 suggest that for music with low subjective beat clarity, it becomes unclear who is moving in synchrony with the auditory stimulus and who is moving asynchronously. Consequently, the context provided by the music becomes less meaningful. In such situations, temporal social entrainment is difficult to assess and affective social entrainment decreases. If the perceived beat clarity is high, temporal social entrainment is easier to assess. In this case, synchronous movements would lead to high affective social entrainment, whereas asynchronous movements would lead to low affective social entrainment.

Although the social entrainment video paradigm enables highly controlled experimental designs, it also has limitations. Walking in synchrony with music is a simple repetitive movement. In the current experiments, each stride clearly fell on the beat in synchronized walking or away from the beat in asynchronized walking. Walking is not a culture-specific movement and the preference for stable phase relationships when walking with another person is found all over the world^3^. The simplicity of the movement and the universal preference for synchronized walking strengthen the internal validity of the current experiments. More complex and culture-specific movements, such as dance, might provide a lower internal but higher external validity. Another limitation is that although the invitation to participate in Study 2 was distributed worldwide, two thirds of the participants were born in Europe or North America. However, by not including music with typical Western structures and instrumentations, we avoided a Western in-cultural bias. Finally, we cannot exclude the possibility that participants were aware of the synchrony manipulation and the corresponding hypothesis. The main effect of synchrony may therefore be influenced by demand characteristics. However, it seems unlikely that demand characteristics affected the more complex interaction effects between synchrony and music ratings, which were the focus of all three studies.

In conclusion, our findings indicate that music can strengthen interpersonal closeness by providing a meaningful context for social interactions, which can increase temporal and affective self-other overlaps. We demonstrate that for this context to become meaningful, a clear perception of the temporal structure of the music is crucial. Without perceiving such a structure, temporal self-other overlaps are reduced and social bonding decreases. Our findings further suggest that the influence of movement synchrony on social bonding during musical activities is less affected by what music we are familiar with but more affected by what music we enjoy. The unique context provided by music can be used to strengthen social bonds that affectively connect people with different backgrounds – especially if these people know how to move in time with the beat and enjoy the same music.

Based on the current findings, future research could investigate whether cultural familiarity with music, enjoyment of music and perceived beat clarity have similar effects on interpersonal closeness in real-life movement interactions. Such studies could also focus on influences of social entrainment with music on prosocial behaviour and communication. Combined with the current findings, research in this direction might provide valuable insights into music and movement supported therapies for individuals with social communication impairments, such as autism spectrum disorders.

## Methods

### Study 1

#### Participants

We collected data from 61 participants (42 female, 19 male, mean age = 22.0 years, *SD* = 3.4) without vision or hearing deficits and with Western cultural backgrounds at the University of Graz, Austria. Two additional participants were excluded because all of their ratings of the dependent variable *inclusion of other in the self* were zero. The Goldsmiths Musical Sophistication Index^48^ indicated that the musical training of the participants was heterogeneous, varying between the 1^st^ and 93^rd^ percentile with a mean at the 36^th^ percentile. Participants provided written informed consent and the study was approved by the ethics committee at the University of Graz. All three studies conform to the code of ethics of the World Medical Association (Declaration of Helsinki).

#### Video stimuli

The 20-second videos can be found in the supplementary material section.

##### Independent variable synchrony

Virtual self and other were either walking in phase with the music (synchronous) or the virtual self was walking in phase and the virtual other out of phase with the music (asynchronous). Each stride consisted of 21 frames. In the synchronous videos, the strides of both figures were occurring at the same frame. In the asynchronous videos, the steps of the virtual other were delayed by eight frames.

##### Independent variable musical pattern

The videos were accompanied by real music with patterns and instrumentations typical for popular North American/Western or Indian music, and by an isochronous metronome. For North American and Indian musical patterns, three instrumental pieces with clear beats were selected (Indian: “Kedara in Vilambit & Drut” by A. A. Khan & T. N. Krishan, “Awakening” by Ken Zuckerman, and “Chaap Tilak” by Shujaat Khan; Western/North American: “What I Got” by Sublime, “Thinking” by The Meters, and “My Father’s Eyes” by Eric Clapton). The tempo of all instrumental pieces (between 92 and 96 bpm in the original versions) was aligned to the stride length of 636 ms/94.3 bpm by using the time warp option in Ableton Live 8 (Ableton, Berlin, Germany). The metronome had an inter-onset-interval of 636 ms.

#### Procedure and ratings

Data were collected in groups of 3 to 4 participants sitting at individual desks with room dividers and wearing closed over-ear headphones. Participants were instructed to watch the stick figure videos and to imagine that they are one of the figures and that the other figure represents an unknown person. They were told that the videos will have different auditory accompaniments and that they should pay attention to how the figures move in time with each other and in time with the auditory accompaniments. Four practice trials with an isochronous metronome as auditory accompaniment were presented at the beginning of the experiment. Afterwards, two blocks with 18 randomized trials were presented – the number of trials followed from the combination of 9 *musical patterns* (3 Indian + 3 North American + 3 metronome) and 2 *synchrony* conditions (synchronous and asynchronous movements).

Participants rated the interpersonal closeness with the virtual other on an adapted Inclusion of Other in the Self scale^32^ (IOS; Fig. 1C) and the *likeability of the other*. The IOS scale is a validated pictorial measure of closeness between self and other, which is not particularly susceptible to social desirability^32^. Both scales were continuous sliders ranging from 0 on the left to 100 on the right. At the end of the experiment, participants rated how much they enjoyed each piece of music and how familiar the music was on a continuous scale from 0 to 100. The experiment lasted approximately 20 minutes.

##### Music ratings

The mean ratings of *familiarity with the music* and *enjoyment of the music* for the 3 Indian and the 3 North American musical stimuli were compared in paired samples t-tests. As expected, *familiarity with the music* was higher for North American compared to Indian music stimuli (*t*(60) = 4.85, *p* < 0.001, *d* = 0.62). Similarly, *enjoyment of the music* was higher for North American compared to Indian music stimuli (*t*(60) = 7.08, *p* < 0.001, *d* = 0.91). A repeated measures correlation (rmcorr package in R) revealed a positive correlation between *familiarity* and *enjoyment* (*r*(314) = 0.34, *p* < 0.001).

### Data analysis

Each participant’s mean rating of *inclusion of other in the self* (IOS) and *likeability of the other* (LIKE) from the three individual stimuli of each *musical pattern* (3 Indian stimuli, 3 North American stimuli, and 3 metronome stimuli) of both blocks were used for the statistical analyses.

We fitted linear mixed effects models to a dataset only including responses to videos with music to explain the dependent variables *inclusion of other in the self* (IOS) and *likeability of the virtual other* (LIKE), using the lmer function from R’s^49^ *lme4* package^50^ (Table 1). The fixed effects of the full models were *synchrony* (synchronous and asynchronous movement), *musical pattern* (familiar/North American and unfamiliar/Indian), *enjoyment of the music*, and the interaction between *synchrony* and *enjoyment of the music*. Based on previous research with a similar design demonstrating the strength of the effect of movement synchrony on affiliation^14^, *synchrony* was tested as first fixed effect. The random effect, noted as (1 | participant), accounted for individual differences by allowing a random intercept per participant. The null-model only included the random effect. The *emmeans* package^51^ in R was used for pairwise comparisons with Bonferroni corrections. A visual data inspection indicated that the residuals of all models were normally distributed. We compared the fit of the nested models using likelihood ratio tests.

### Study 2

#### Participants

Participants between the ages of 18 and 60 without vision or hearing deficits were recruited over mailing lists and social media. Out of 271 participants who started the survey, 204 completed every question and were included in the analysis (112 female, 92 male, mean age = 36.0 years, *SD* = 10.9). The participants were born in Europe: 114, Asia: 41, North America: 21, Latin America: 14, Africa: 8, and Oceania: 6. According to the Goldsmiths Musical Sophistication Index^48^ the musical training of the participants was heterogeneous, varying between the 1^st^ and 99^th^ percentile with a mean at the 54^th^ percentile. 141 participants used a laptop or computer (92 with headphones, 29 with external loudspeakers, and 20 with integrated loudspeakers) and 63 used a smartphone or tablet (31 with headphones, 8 with external loudspeakers, and 24 with integrated loudspeakers). Written informed consent was provided and the study was approved by the institutional review board at the Danish Neuroscience Centre.

#### Video stimuli

The 14-second videos can be found in the supplementary material section.

##### Independent variable synchrony

Virtual self and other were either walking in synchrony with each other and the music (beat interval: 700 ms/85.7 bpm) or the virtual other was walking asynchronously. In the synchronous movement videos, the step frequency of the virtual other was slowed down by 1%, i.e., 693 ms and the phase was slightly shifted. As a result, the steps of the two figures were approximately 60 ms apart at the beginning of the video, perfectly synchronized in the middle of the video, and approximately 60 ms apart in the end of the video introducing barely noticeable “human-like” imperfections. In the asynchronous movement videos, the virtual other was not only walking out of phase with the beat but also with a different step frequency, i.e., 800 ms instead of 700 ms.

##### Independent variable musical pattern

Based on Pre-studies 2A and 2B (see supplementary material), the following three musical stimuli were selected for the main experiment: “Bonde” by Ali Farka Toure and Ry Cooder from the album “Talking Timbuktu” (region: West Africa, 82 bpm), “Cumbia del Leon” by The Lions from the album “Jungle Struttin” (region: Latin America, 84 bpm), and “Nomads” by Zakir Hussain from the album “Music of the Deserts” (region: South Asia, 85 bpm). The outcomes of Prestudies 2A and 2B for these stimuli are presented in Table 4.

**Table 4:** Descriptive statistics of the three music stimuli selected after Pre-studies 2 A and 2B. Results of the finger tapping task (mean and standard deviation of inter-tap-intervals; beat interval: 700 ms) and the synchrony rating in which participants had to decide if a stick figure was walking in time with the beat of the music or out of time (percentage of correct answers and mean of the time needed for a decision).

Stimulus	Region	Pre-study 2 A: Online rating			Pre-study 2B: Finger tapping			Pre-study 2B: Synchrony rating		
		Enjoy *(SD)*	Mood *(SD)*	Familiar *(SD)*	Correct origin %	Mean of ITIs (SD)	SD of ITIs (SD)	Number of taps (SD)	% of correct answers (*SD*)	Decision time in sec (*SD*)
Bonde	West Africa	5.4 (1.3)	5.5 (1.0)	3.7 (1.5)	39	755 (144)	65 (42)	14.6 (3.0)	73 (23)	4.19 (1.06)
Cumbia del Leon	Latin America	5.9 (2.1)	6.1 (1.7)	5.4 (2.2)	55	716 (51)	28 (7)	16.2 (1.6)	83 (22)	3.78 (0.98)
Nomads	South Asia	5.6 (1.8)	5.6 (1.4)	4.9 (1.9)	50	730 (90)	37 (19)	15.7 (1.7)	83 (19)	3.76 (0.82)

#### Survey and ratings

The survey was carried out online on soscisurvey.de (SoSci Survey GmbH, Munich, Germany). A one-minute instruction video explained the task and the rating scales. After the instructions, six videos resulting from the combination of the independent variables (2 *synchrony* × 3 *musical patterns*) were presented. Participants rated the social closeness between the virtual self and other on an adapted Inclusion of Other in the Self scale^32^ (IOS) with a continuous slider ranging from zero on the left end to 100 on the right end (Fig. 1C). In contrast to Study 1, we did not include ratings of the likeability of the virtual other for two reasons. First, IOS ratings seem to better reflect the relevant social processes and evaluations in the current paradigm, while ratings of the likeability of the other might have been confounded with the liking of the music. Second, we reduced the duration of the online experiment to reach more participants.

The videos were presented in synchronous/asynchronous pairs per *musical pattern*. The order of *musical patterns* and the order of the movement condition within a *musical pattern* were randomized. After completing the video ratings, participants rated the music without any visual stimulus on the following continuous scales from 0 (“not at all”) to 100 (“very”): “How familiar are you with this general type of music?”, “How much did you like this specific piece of music”, and “How clear was the beat of this specific piece of music?”. Finally, participants filled out the musical training subscale of the Goldsmith Musical Sophistication Index^48^. The whole survey took approximately 10 minutes.

##### Music ratings

We analysed the *familiarity with the music*, the *enjoyment of the music*, and the perceived *beat clarity of the music* in three separate one-way repeated measures ANOVAs in the software JASP with the factor *musical pattern* (West Africa, South Asia, and Latin America). Greenhouse-Geisser corrections were applied when the assumption of sphericity was violated. Post-hoc comparisons were Bonferroni corrected. *Familiarity with the music* significantly differed between the three *musical patterns* (*F*(2,406) = 28.66, *p* < 0.001, *η*² = 0.12), with Latin American stimulus rated as more familiar than West African (mean difference = 12.74, *SE* = 2.10, *p* < 0.001, *d* = 0.43) and South Indian stimuli (mean difference = 14.53, *SE* = 2.23, *p* < 0.001, *d* = 0.46) and no difference between the latter. *Enjoyment of the music* did not significantly differ between the three *musical patterns* (*F*(2,406) = 2.35, *p* = 0.097, *η*² = 0.01). Perceived *beat clarity of the music* significantly differed between the three *musical patterns* (*F*(1.88,381.64) = 38.66, *p* < 0.001, *η*² = 0.16). The beat of the Latin American stimulus was perceived as clearer than the beat of the South Asian (mean difference = 3.41, *SE* = 1.41, *p* = 0.050, *d* = 0.17) and West African stimuli (mean difference = 13.78, *SE* = 1.72, *p* < 0.001, *d* = 0.56). Additionally, the beat of the South Asian stimulus was perceived as clearer than the beat of the West African stimulus (mean difference = 10.37, *SE* = 1.75, *p* < 0.001, *d* = 0.42). Within each *musical pattern*, *familiarity with the music*, *enjoyment of the music*, and perceived *beat clarity* were positively correlated with each other (all *r*(202)> 0.21, all *p* < 0.002). As shown in Supplementary Table S6, *familiarity*, *enjoyment*, and *beat clarity* ratings for the selected music stimuli were relatively homogenous between participants born in the following regions: West Africa, Latin America, South Asia, and others.

#### Data analysis

By using the lmer function from R’s^49^ *lme4* package^50^, we modelled IOS as a function of the following effects in the full models: *synchrony*, *musical pattern*, *music rating* (either *familiarity with the music*, *enjoyment of the music*, or perceived *beat clarity of the music*), and the interaction between *synchrony* and *music rating* (Table 2). Additionally, the random effect (1 | participant) accounted for individual differences by allowing a random intercept per participant. The null-model only included the random effect. The *emmeans* package^51^ in R was used for pairwise comparisons with Bonferroni corrections. A visual data inspection indicated that the residuals of the models were normally distributed. We compared the fit of the nested models using likelihood ratio tests.

### Study 3

#### Participants

Forty-eight students at Aarhus University enrolled on a variety of study programs took part in the study (35 female, 13 male, mean age = 23.4 years, *SD* = 3.5). Data were collected in group tests with 26 and 22 participants, at the beginning of two separate lectures. Participation was voluntary and informed consent was provided by optionally returning the paper-and-pencil questionnaires that did not contain identifying information. The study was approved by the institutional review board at the Danish Neuroscience Centre.

#### Video stimuli

The 12-second videos can be found in the supplementary material section.

##### Independent variable synchrony

Virtual self and other were either walking in synchrony with each other and the music or the virtual other was walking asynchronously. The step frequency of both figures in the synchronous movement condition was 636 ms (94.4 bpm). The step frequency of the virtual other in the asynchronous movement condition was 700 ms.

##### Independent variable syncopation level

The three auditory stimuli were taken from a larger set of stimuli used in Matthews, Witek, Heggli, Penhune, and Vuust^41^. They consisted of repetitions of five identical piano chords in D major and a soft hi-hat sound marking the eighth notes. The stimuli had three syncopation levels: Low, moderate, and high (Fig. 3A), related to low, moderate, and high beat clarity, respectively. The sequences were slowed down to 94.4 bpm.

#### Procedure and ratings

Data were collected in two group sessions at the beginning of two different lectures, which were part of different lecture series at Aarhus University. Participants received a printed questionnaire with instructions on the first page. The instructions were additionally read aloud by the experimenter. The videos were presented on a screen with a beamer. Sound was played via active loudspeakers. The experimental stimuli consisted of six individual videos, resulting from the combination of the independent variables *synchrony* (2) × *syncopation level* (3). Each video was presented twice in two separate blocks resulting in 12 trials. The order of the six videos per block was randomized. After each video, participants had a few seconds to provide and answer on an adapted Inclusion of Other in the Self scale^32^ (IOS; Fig. 1C), which was printed on the questionnaire including a visual-analogue scale with a length of 100 mm, corresponding to IOS values of 0 to 100, similar to Studies 1 and 2. The experiment lasted approximately 10 minutes.

#### Data analysis

As a visual data inspection and Shapiro-Wilk normality tests indicated that most of the IOS distributions were not normal, we used Wilcoxon signed-rank tests for the analysis and computed the effect size as *r* = *Z*/sqrt(N × 2) with N = 48. The Bonferroni-corrected critical p-value for the resulting six comparisons is 0.05/6 = 0.0083. Additionally, we computed and compared IOS difference values (synchronous movement – asynchronous movement) for every syncopation level.

## Supplementary information

Supplementary Information.

Supplementary Information 2.

Supplementary Information 3.

Supplementary Information 4.

## Data Availability

The data supporting the findings of these studies are available from the corresponding author upon request.
