# Supplementary material for: Cultural Familiarity and Individual Musical Taste Differently Affect Social Bonding when Moving to Music
Source: Sci Rep. 2020 Jun 22;10:10015. doi: 10.1038/s41598-020-66529-1 (PMC7308378; doi:10.1038/s41598-020-66529-1)
Supplement: Supplementary file 4 — Supplementary Information 4. [file 41598_2020_66529_MOESM4_ESM.pdf]

Supplementary Material

Cultural Familiarity and Individual Musical Taste Differently Affect Social Bonding when Moving to Music

Jan Stupacher[1,2], Maria A. G. Witek[3], Jonna K. Vuoskoski[4], Peter Vuust[1]

[1] Center for Music in the Brain, Department of Clinical Medicine, Aarhus University & The Royal Academy of Music Aarhus/Aalborg, Denmark

[2] Institute of Psychology, University of Graz, Austria

[3] Department of Music, University of Birmingham, United Kingdom

[4] RITMO Centre for Interdisciplinary Studies in Rhythm, Time and Movement, Department of Musicology & Department of Psychology, University of Oslo, Norway

Corresponding author:

Jan Stupacher
Center for Music in the Brain
Aarhus University Hospital, Nørrebrogade 44
8000 Aarhus C, Denmark

Email: stupacher@clin.au.dk

Study 1: Music vs. Metronome in Social Entrainment – ANOVAs

We computed two 2×3 repeated measures ANOVAs in the software JASP, one on the *inclusion of other in the self* ratings (IOS) and one on the *likeability of the other* ratings (LIKE). The factors were *synchrony* (synchronous vs. asynchronous movement) and *musical pattern* (culturally familiar music vs. culturally unfamiliar music vs. metronome). Greenhouse-Geisser corrections were applied when the assumption of sphericity was violated. Post-hoc comparisons were Bonferroni corrected.

The ANOVA on IOS ratings revealed significant main effects of *synchrony* ($F(1,60) = 139.23$, $p < .001$, $\eta^2 = .70$) and *musical pattern* ($F(2,120) = 45.59$, $p < .001$, $\eta^2 = .43$), but no significant interaction between the two factors ($F(1.78,106.68) = 0.01$, $p = .992$, $\eta^2 = .00$). IOS was rated higher with synchronous compared to asynchronous movements (mean difference = 27.84, $SE = 2.36$, $p < .001$, $d = 1.51$), higher for culturally familiar music compared to culturally unfamiliar music (mean difference = 5.94, $SE = 1.19$, $p < .001$, $d = 0.64$) and the metronome (mean difference = 12.88, $SE = 1.52$, $p < .001$, $d = 1.09$), and higher for culturally unfamiliar music compared to the metronome (mean difference = 6.94, $SE = 1.32$, $p < .001$, $d = 0.67$).

The ANOVA on LIKE ratings revealed significant main effects of *synchrony* ($F(1,60) = 79.54$, $p < .001$, $\eta^2 = .57$) and *musical pattern* ($F(1.81,108.83) = 50.11$, $p < .001$, $\eta^2 = .46$), but no significant interaction between the two factors ($F(2,120) = 0.89$, $p = .412$, $\eta^2 = .02$). LIKE was rated higher with synchronous compared to asynchronous movements (mean difference = 17.27, $SE = 1.94$, $p < .001$, $d = 1.14$), higher for culturally familiar music compared to culturally unfamiliar music (mean difference = 8.33, $SE = 1.42$, $p < .001$, $d = 0.75$) and the metronome (mean difference = 17.18, $SE = 1.89$, $p < .001$, $d = 1.17$), and higher for culturally unfamiliar music compared to the metronome (mean difference = 8.84, $SE = 1.81$, $p < .001$, $d = 0.63$).

Pre-study 2A: Stimulus Selection

Twelve instrumental music stimuli of 14 s duration were selected for Pre-study 2A. Four musical pieces were considered typical for the broad topographical regions West Africa, Latin America, and South Asia, respectively (Table S3). The music stimuli were slightly time stretched (without any pitch changes) by using the time warp option in Ableton Live 8 to fit the tempo of 85.7 beats per minute / 700 ms quarter beat interval. To evaluate the preselected stimuli, 59 participants (40 female, 18 male, 1 other; mean age 27.0 years, $SD = 5.3$) from 33 countries (39 Europe, 7 Asia, 5 North America, 4 South America, 4 Africa) completed an online questionnaire. Participants rated the familiarity with the type of music (very unfamiliar – very familiar), the enjoyment of the specific music clip (did not enjoy – enjoyed very much), and the mood elicited by the specific music clip (very negative mood – very positive mood) on 9-point Likert scales. Additionally, participants indicated where they thought the music comes from in a forced-choice question with the following options: Northern America, Southern America, Europe, Africa, Northern Asia, Southern Asia, or Oceania. The geographic regions were displayed on a world map for orientation. The six music stimuli with the most similar ratings (two from each geographical region) were selected for further evaluation (Table S4).

Pre-study 2B: Stimulus Selection

The second pre-study was designed to evaluate the perceived beat clarity of the music stimuli selected in Pre-study 2A. It consisted of two tasks that were counterbalanced between 12 European participants (7 female, 5 male, mean age 24.9 years, $SD = 3.8$).

Task 1: Finger tapping. Participants were asked to tap their right index finger as accurately as possible in time with the beat of the six music stimuli selected in Pre-study 2A. Each stimulus lasted 14 s and was presented four times in separate blocks resulting in 24 trials overall. Max/MSP (Cycling '74) was used to present the music stimuli and to record the taps. Participants tapped on a 3 x 3 cm aluminum pad connected to an Arduino based circuit board (MaKey MaKey, JoyLabz). As dependent variables, the number of taps, means and standard deviations of inter-tap-intervals (ITIs) for each stimulus were computed. ITIs were calculated as the difference between the absolute time of a tap n and the absolute time of the following tap n+1 in milliseconds. At least eight taps had to be recorded for a trial to be included in the analysis (4 of 288 trials excluded). ITIs smaller than 0.375 times the 700 ms beat interval of the music stimuli and longer than 2.25 times the beat interval were excluded. This liberal time window was used to include potential taps at the eighth note (350 ms) and half note level (1400 ms). Additionally, taps with ITIs bigger or smaller than two standard deviations from the mean ITI for each participant and trial were excluded (4.96 %). Descriptive statistics of all six music stimuli can be found in Table S5.

Task 2: Synchrony rating. In a forced choice paradigm, participants had to decide whether a single stick figure was walking in time with the beat of a music clip or out of time. Participants were told to answer as fast as possible, but also as correct as possible. The music stimuli were shortened versions of the six music stimuli used in the finger-tapping task. The clips were cut in the beginning to ensure that after a short fade-in, they would all start on a downbeat and with a step in time with this downbeat. In the synchronous videos, the step frequency was the same as the tempo of the musical patterns (700 ms). Consequently, the single steps were aligned with the quarter beat of the music. In the asynchronous videos, the step frequency was 800 ms, resulting in unaligned steps and quarter beats. The combination of independent variables resulted in 12 videos in total (6 music stimuli, 2 synchrony conditions). Each video lasted 10 s and was presented twice in separate blocks, resulting in 24 trials overall. Answers provided between 1 and 10 s after stimulus onset were included in the analysis (7 of 288 answers fell outside this time window). Descriptive statistics for all videos can be found in Table S5.

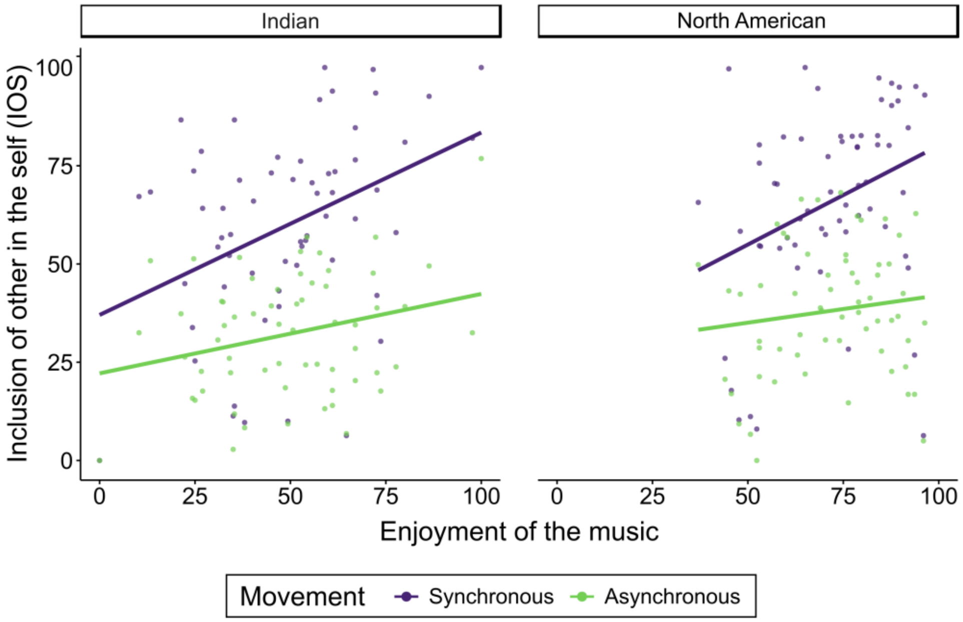

Figure S1. Data points and regression lines for ratings of interpersonal closeness (as measured by the IOS scale) in relation to ratings of the enjoyment of the music in Study 1. IOS ratings of videos with Indian musical patterns (unfamiliar) are displayed on the left side and IOS ratings of videos with North American musical patterns (familiar) are displayed on the right side.

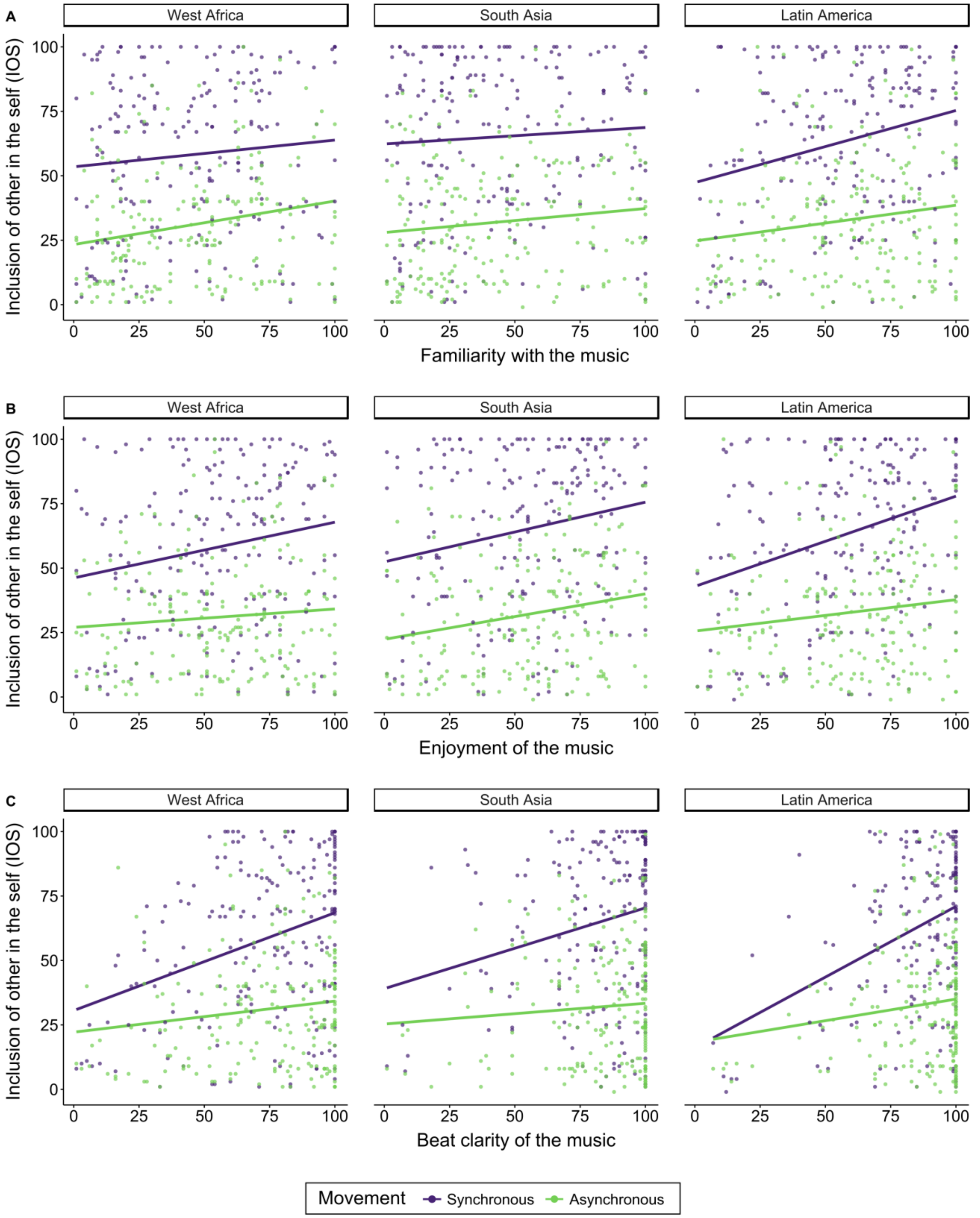

Figure S2. Data points and regression lines for ratings of interpersonal closeness (as measured by the IOS scale) in relation to ratings of (A) familiarity with the music, (B) enjoyment of the music, and (C) the perceived beat clarity in Study 2. From left to right, the figures depict IOS ratings of videos with West African, South Asian, and Latin American musical patterns.

Table S1. Pairwise comparisons of individual factor levels in linear mixed effects models of Study 1. Comparisons were computed using the *emmeans* package in R.

**Model**	**Pairwise comparison**	**Estimate**	***SE***	***df***	***t***	***p****	**95% CI***
IOS ~ Synchrony + Musical Pattern	Synchronous – Asynchronous	27.80	1.55	181	17.99	< .001	[24.31, 31.29]
	Unfamiliar Music – Familiar Music	-5.94	1.55	181	-3.84	< .001	[-9.43, -2.44]
IOS ~ Synchrony × Enjoyment + Musical Pattern	Synchronous – Asynchronous	27.80	1.48	179	18.76	< .001	[24.22, 31.38]
	Unfamiliar Music – Familiar Music	-1.24	1.95	197	-0.63	1	[-5.95, 3.48]
	Slope Enjoyment | Sync – Enjoyment | Async	0.22	0.07	179	3.11	.007	[0.05, 0.39]
LIKE ~ Synchrony + Musical Pattern	Synchronous – Asynchronous	16.99	1.42	181	11.93	< .001	[13.80, 20.21]
	Unfamiliar Music – Familiar Music	-8.33	1.42	181	-5.88	< .001	[-11.60, -5.11]
LIKE ~ Synchrony + Musical Pattern + Enjoyment	Synchronous – Asynchronous	17.00	1.35	177	12.58	< .001	[13.73, 20.26]
	Unfamiliar Music – Familiar Music	-0.67	1.73	210	-0.39	1	[-4.85, 3.51]
	Slope Enjoyment	0.34	0.05	239	7.08	< .001	[0.23, 0.46]

* Bonferroni corrected

Table S2. Pairwise comparisons of individual factor levels in linear mixed effects models of Study 2. Comparisons were computed using the *emmeans* package in R.

**Model**	**Pairwise comparison**	**Estimate**	***SE***	***df***	***t***	***p*** *	**95% CI** *
IOS ~ Synchrony + Musical Pattern + Familiarity	Synchronous – Asynchronous	30.36	1.09	1016	27.81	< .001	[27.54, 33.18]
	West Africa – South Asia	-4.20	1.34	1017	-3.14	.009	[-7.65, -0.74]
	West Africa – Latin America	-2.28	1.38	1044	-1.65	.498	[-5.85, 1.29]
	South Asia – Latin America	1.92	1.40	1052	1.37	.855	[-1.69, 5.52]
	Slope Familiarity	0.10	0.03	1201	3.70	.001	[0.03, 0.18]
IOS ~ Synchrony × Enjoyment + Musical Pattern	Synchronous – Asynchronous	30.36	1.08	1015	28.18	< .001	[27.58, 33.14]
	West Africa – South Asia	-4.11	1.32	1015	-3.12	.009	[-7.52, -0.71]
	West Africa – Latin America	-3.02	1.32	1017	-2.28	.114	[-6.43, 0.40]
	South Asia – Latin America	1.10	1.32	1018	0.83	1	[-2.32, 4.52]
	Slope Enjoyment | Sync – Enjoyment | Async	0.14	0.04	1015	3.49	.003	[0.04, 0.25]
IOS ~ Synchrony × Beat Clarity + Musical Pattern	Synchronous – Asynchronous	30.36	1.06	1015	28.61	< .001	[27.62, 33.10]
	West Africa – South Asia	-1.62	1.35	1046	-1.20	1	[-5.10, 1.87]
	West Africa – Latin America	-0.43	1.39	1066	-0.31	1	[-4.01, 3.15]
	South Asia – Latin America	1.19	1.31	1019	0.91	1	[-2.18, 4.55]
	Slope Beat Clarity | Sync – Beat Clarity | Async	0.28	0.05	1015	6.14	< .001	[0.16, 0.40]

* Bonferroni corrected

Table S3. List of songs included in Pre-study 2A. For every song, we changed the tempo to 85.7 beats per minute (700 ms) and cut a short instrumental clip of 13 seconds.

Song name	Artist(s)	Region	Code
Kèlè	Fatoumata Diawara	West Africa	WA1
Allah Uya	Ali Farka Toure	West Africa	WA2
A.S.C.O.	Ali Farka Toure	West Africa	WA3
Bonde	Ali Farka Toure and Ry Cooder	West Africa	WA4
Chin Chon Chow	Louie Ramirez	Latin America	LA1
Bongo	Calambuco	Latin America	LA2
Cumbia del Leon	The Lions	Latin America	LA3
Guateque Campesino	Ibrahim Ferrer	Latin America	LA4
Chandan Sa Badan	Rakesh Chaurasia	South Asia	SA1
The Great Indian Desert	Zakir Hussain	South Asia	SA2
Nomads	Zakir Hussain	South Asia	SA3
Awakening	Ken Zuckerman	South Asia	SA4

Table S4. Results of familiarity, enjoyment, and mood / valence ratings of Pre-study 2A. Stimuli marked with an asterisk were selected for Pre-study 2B. The primary selection criteria were correct answers for region of origin and ratings of enjoyment. Stimulus codes: West Africa (WA), Latin America (LA), and South Asia (SA).

**Stimulus code**	**Familiarity**	**Enjoyment**	**Mood**	**Correct origin in %**
WA1	5.29 (1.92)	6.10 (1.78)	6.26 (1.57)	6.5
WA2*	4.22 (2.36)	5.63 (1.78)	6.15 (1.20)	11.1
WA3	4.90 (2.04)	6.45 (1.86)	6.48 (1.41)	12.9
WA4*	3.68 (1.54)	5.36 (1.34)	5.46 (1.04)	39.3
LA1*	5.94 (1.91)	6.32 (1.87)	6.58 (1.50)	54.8
LA2	6.89 (1.40)	7.11 (1.31)	7.61 (1.03)	85.7
LA3*	5.35 (2.17)	5.94 (2.05)	6.06 (1.71)	54.8
LA4	5.93 (1.78)	7.18 (1.36)	7.21 (1.13)	57.1
SA1	5.04 (2.49)	5.71 (2.24)	5.61 (1.42)	64.3
SA2	5.06 (2.29)	5.03 (2.14)	5.71 (1.44)	45.2
SA3*	4.86 (1.90)	5.57 (1.77)	5.64 (1.37)	50.0
SA4*	5.26 (1.59)	6.13 (1.80)	6.16 (1.55)	41.9

* Selected for Pre-study 2B

Table S5. Results of the two behavioral tasks of Pre-study 2B. The inter-beat-interval of all musical stimuli was 700 ms. Taking into account the results of Pre-study 2A, the stimuli marked with an asterisk were selected for the main study. Stimulus codes: West Africa (WA), Latin America (LA), and South Asia (SA).

	**Pre-study 2B: Finger tapping**			**Pre-study 2B: Synchrony rating**	
Stimulus	Mean of ITIs (SD)	SD of ITIs (SD)	Number of taps	% of correct answers (SD)	Decision time in sec (SD)
WA2	767 (137)	49 (28)	14.5 (1.9)	88 (23)	3.96 (0.88)
WA4*	755 (144)	65 (42)	14.6 (3.0)	73 (23)	4.19 (1.06)
LA1	706 (53)	44 (22)	16.4 (2.0)	84 (26)	4.16 (1.04)
LA3*	716 (51)	28 (7)	16.2 (1.6)	83 (22)	3.78 (0.98)
SA3*	730 (90)	37 (19)	15.7 (1.7)	83 (19)	3.76 (0.82)
SA4	743 (96)	61 (36)	15.2 (1.8)	73 (31)	4.62 (1.52)

* Selected for Study 2

Table S6. Mean (SD) familiarity and enjoyment ratings separated for participants region of origin (West Africa, N = 8; South Asia, N = 41; Latin America, N = 14; Others, N = 141).

	**Region of origin**			
	West Africa	South Asia	Latin America	Others
Familiarity with West African music stimulus	37.6 (32.2)	58.4 (26.9)	34.8 (33.2)	42.9 (25.7)
Familiarity with South Asian music stimulus	34.9 (24.6)	62.4 (34.9)	36.9 (33.9)	39.1 (26.1)
Familiarity with Latin American music stimulus	65.0 (29.3)	55.6 (26.7)	68.1 (35.9)	57.3 (26.6)
Enjoyment of West African music stimulus	63.4 (34.9)	61.1 (25.9)	46.8 (34.1)	54.1 (25.5)
Enjoyment of South Asian music stimulus	57.0 (35.6)	61.8 (26.8)	48.5 (29.9)	53.1 (25.6)
Enjoyment of Latin American music stimulus	66.9 (31.5)	57.8 (21.0)	60.7 (36.1)	58.7 (26.7)
Beat clarity of West African music stimulus	90.9 (18.0)	72.9 (25.8)	62.6 (34.1)	72.6 (25.1)
Beat clarity of South Asian music stimulus	95.1 (5.6)	76.2 (26.8)	79.9 (24.8)	84.7 (18.9)
Beat clarity of Latin American music stimulus	95.8 (6.0)	79.8 (24.6)	76.9 (28.3)	88.9 (15.8)
